# Identification and Validation of Reference Genes for Transcript Normalization in Strawberry (*Fragaria* × *ananassa*) Defense Responses

**DOI:** 10.1371/journal.pone.0070603

**Published:** 2013-08-05

**Authors:** Francisco Amil-Ruiz, José Garrido-Gala, Rosario Blanco-Portales, Kevin M. Folta, Juan Muñoz-Blanco, José L. Caballero

**Affiliations:** 1 Departamento de Bioquímica y Biología Molecular e Instituto Andaluz de Biotecnología, Campus Universitario de Rabanales y Campus de Excelencia Internacional Agroalimentario-CEIA3, Universidad de Córdoba, Córdoba, Andalucía, Spain; 2 Horticultural Sciences Department and The Graduate Program for Plant Molecular and Cellular Biology, University of Florida, Gainesville, Florida, United States of America; Instituto de Biología Molecular y Celular de Plantas, Spain

## Abstract

Strawberry (*Fragaria spp*) is an emerging model for the development of basic genomics and recombinant DNA studies among rosaceous crops. Functional genomic and molecular studies involve relative quantification of gene expression under experimental conditions of interest. Accuracy and reliability are dependent upon the choice of an optimal reference control transcript. There is no information available on validated endogenous reference genes for use in studies testing strawberry-pathogen interactions. Thirteen potential pre-selected strawberry reference genes were tested against different tissues, strawberry cultivars, biotic stresses, ripening and senescent conditions, and SA/JA treatments. Evaluation of reference candidate’s suitability was analyzed by five different methodologies, and information was merged to identify best reference transcripts. A combination of all five methods was used for selective classification of reference genes. The resulting superior reference genes, *FaRIB413, FaACTIN, FaEF1α and FaGAPDH2* are strongly recommended as control genes for relative quantification of gene expression in strawberry. This report constitutes the first systematic study to identify and validate optimal reference genes for accurate normalization of gene expression in strawberry plant defense response studies.

## Introduction

Transcriptomic analyses are essential in understanding complex molecular processes occurring in plants. Although global evaluation techniques such as microarrays or RNAseq provide a representative snapshot of a transcriptome, these techniques can only be practically applied to a limited number of tissues, treatments or time points. The data found by global expression techniques need to be then considered carefully, typically using relative quantification of gene expression by quantitative reverse transcription (RTqPCR). This method is used as a primary source of in-depth molecular expression information for a smaller set of gene candidates due to its wide range of quantification, reproducibility, and higher precision and accuracy [1], [2], [3]. However, this approach requires knowledge of stably expressed reference genes for data normalization of target genes under specific experimental conditions. Failure to use an appropriate reference or internal control gene may result in biased gene expression profiles, as well as low reproducibility. Consequently, either only gross changes in gene expression level are declared statistically significant, or the pattern of gene expression is inaccurately characterized [4], [5].

To date, some of the best known and most frequently used reference gene transcripts for RTqPCR in plants and animals include those coding for 18S rRNA, glyceraldehyde-3-phosphate dehydrogenase, elongation factor-1α, actin, and α- and β-tubulin [6], [7], [8], [9], [10], [11], [12], [13]. These genes have been recognized as stably expressed housekeeping genes, and they have been historically used as reference genes in many plants when normalizing RNA-gel blots and semi-quantitative PCR. However, numerous reports have indicated that transcript accumulation is not always consistent under some experimental conditions or across tissues. Such variation, may introduce a significant level of error in interpreting the actual expression pattern of a target gene [14], [15]. Identification of most appropriate and highly-stable internal reference genes for normalization in any given experimental plant system is a prerequisite and compulsory step to obtain reliable and reproducible results from RTqPCR. A strong reference is the foundation of accurate RTqPCR analyses following the golden rules which have been detailed recently in Udvardi et al. [16].

Over the last few years efforts have been made to identify suitable reference genes for quantification of gene expression in model plant species such as Arabidopsis [17]. Efforts have been extended to crop plants such as pea [18], banana [19], [20], sulla [21], zucchini [22], and citrus [23]. However, reference genes still have yet been identified and tested in other species of high agricultural interest including strawberry (*Fragaria spp*), a small fruit crop of great value throughout the world (FAOSTAT Agriculture Data [http://faostat.fao.org/, updated 7 aug 2012]).

Due to its broad horticultural importance and relatively close relationship to other valuable rosaceous crops, strawberry has been proposed as a model for functional genomics and transgenic studies within the Rosaceae [24], [25]. Strawberry’s rapid cycling, fast growth and relative transformability make it an attractive system for functional evaluation of genes associated with plant traits not testable in Arabidopsis. An increasing number of molecular studies are being reported in this species. Many of these studies have performed RTqPCR analysis using traditional reference genes to describe a wide variety of molecular events occurring in strawberry. The technique has been used to query gene expression during plant development, fruit ripening, aroma production, and responses to many biotic and abiotic stresses [26], [27], [28], [29], [30]. However, the suite of strawberry reference genes has not been carefully vetted to determine their optimal suitability for comparative expression analyses across a range of conditions, tissues or treatments.

It is necessary to identify candidate genes specifically chosen for transcript normalization for the conditions under study [31], [32]. Also, when using only one reference gene, its stability cannot be properly evaluated. The use of multiple reference genes does not only produce more reliable data, it permits an internal evaluation of the stability of these reference transcripts as well.

In the present study a subset of candidate reference genes for strawberry RTqPCR normalization in plant defense studies were identified and tested. Candidates were evaluated across a range of forty-eight situations distributed over seven experimental conditions including fruit ripening stages, biotic stress after *Colletotrichum acutatum* infection, and treatments with plant hormones such as SA and MeJA. Also, different cultivars of strawberry (*Fragaria × ananassa*), and growth conditions were tested. Recommendations for the use of these candidate genes are provided to ensure an accurate normalization of transcript level under a given condition in strawberry gene expression studies by RTqPCR.

## Results

### Selection of Candidate Reference Genes in Strawberry for Gene Expression Analysis

Candidate genes were selected for further analysis based on in-house data and information obtained from a range of microarrays experiments ([33], Amil-Ruiz et al., unpublished). Specific strawberry transcripts have been identified that exhibit a high degree of stability among biological replicates and in varying experimental conditions. Due to the fact that low abundance transcripts generally show high variation in their basal expression [34] they were not considered further. The analysis was performed only with candidates whose primers match prescribed conditions described below. In addition, the analysis sought to examine transcripts representing a cross-section of functional diversity to avoid a putative co-regulation effect among genes that may respond in parallel in response to a particular experimental assay. Such precautions are a prerequisite for one of the statistical procedures (the geNORM algorithm) reported to identify stably expressed genes [4].

Under all of these restrictive conditions, thirteen preselected candidate genes were identified (Table 1). These genes encode molecular components associated with a wide variety of biological functions in plant cell physiology such as 18S rRNA (gene *FaRIB413*), a ribosome component; *GLYCERALDEHYDE-3-PHOSPHATE DEHYDROGENASE* (genes *FaGAPDH1* and *FaGAPDH2*), an essential enzyme for carbohydrate metabolism in cytoplasm; *ELONGATION FACTOR*-1α (*gene FaEF1α*), a component of the protein synthesis machinery; *ACTIN* (gene *FaACTIN*), α-*TUBULIN* (gene *FaTUBα*) and β-*TUBULIN* (gene *FaTUBβ*), major components of microfilament and microtubule of the cytoskeleton, respectively; the *UBIQUTIN CONJUGATING ENZYME* E2 (gene *FaUBQ1*), a basic component of the ubiquitin-mediated protein tagging system; chromatin remodeling protein *CHC1* (gene *FaCHC1*), an essential part of the chromodomain remodeling complex; an S-adenosyl-L-methionine-dependent methyltransferase (gene *FaMT1*), an enzyme implicated in secondary metabolism; a strawberry ortholog of the Arabidopsis *AtBZIP61* regulatory transcription factor (gene *FaBZIP1*); a mitochondrial import inner membrane translocase (gene *FaTIM1*); a protein with a forkhead-associated domain and unknown molecular function (gene *FaFHA1*). In addition, the *FaWRKY1* gene, a previously reported strawberry gene known to respond to all the different biological conditions used in this study [30], was chosen as a target gene to test the validity of these strawberry candidate genes as good reference genes in RTqPCR analyses.

**Table 1:** Information of selected genes after evaluation, and characteristics of PCR products and primers used in this analysis.

Fragaria x ananassagene ID	Fragaria vesca orthologe (a)	Gene description	Oligo orientation	Sequence (5′-3′)	Primer melting temp(°C) (b)	Product size (bp)	Optimal anealing (°C) (c)	PCR product melting temp (°C) (d)	PCR efficiency ± SD (e)	Ref (f)
FaGAPDH2	gene07104	Glyceraldehyde-3-phosphate dehydrogenase	sense chain	CCCAAGTAAGGATGCCCCCATGTTCG	82,1	117	65	85	1,769±0,028	[26]
			anti-sense chain	TTGGCAAGGGGAGCAAGACAGTTGGTAG	81,2					
FaUBQ1	gene08438	Ubiquitin E2	sense chain	CCCATCTCCGACAACCGCCACATCTAAA	83,1	130	66	89,5	1,727±0,028	
			anti-sense chain	CCGCCGCCCCAATCTTCTGTACTCC	82,7					
FaGAPDH1	gene18492	Glyceraldehyde-3-phosphate dehydrogenase	sense chain	GGCTTCTATCTCAACCGGCTCGTCTT	77,7	121	65	85	1,925±0,025	[51]
			anti-sense chain	CTTCCCACTGCTCCCTGATCTCTGATAC	77,3					
FaEF1α	gene28639, gene28622, gene23217	Elongation factor 1-alpha	sense chain	TGGATTTGAGGGTGACAACATGA	73,1	145	65	87	1,798±0,028	[27]
			anti-sense chain	GTATACATCCTGAAGTGGTAGACGGAGG	73,3					
FaTUBα	gene01798, gene05604, gene03851, gene26908	Tubulin alpha	sense chain	CATGGCTTGCTGTTTGATGTACCGTG	78,5	156	65	87	1,765±0,027	
			anti-sense chain	GGGACAACAGTGGGTGGCTGGTAGTT	79,1					
FaTUBβ	gene07781, gene13266, gene20192, gene08531, gene18775	Tubulin beta	sense chain	ACACTGTTGTGGAGCCTTACAATGCTAC	74,7	172	65	85,5	1,773±0,024	
			anti-sense chain	GACATTGTTGCGGAGATCAAGTGATT	74,8					
FaACTIN	gene26612, gene18390, gene22626, gene18570, gene14112, gene01836	Actin	sense chain	GGGCCAGAAAGATGCTTATGTCGG	77	152	65	85	1,801±0,029	[28]
			anti-sense chain	GGGCAACACGAAGCTCATTGTAGAAG	76,2					
FaCHC1	gene25887	SWI/SNF complex component	sense chain	CATCTGTTTCCGCCACAACCTATACAT	75,1	156	65	83,5	1,762±0,03	
			anti-sense chain	TTTGTTTTTCTCTGAGTTGGCCATTAGA	74,1					
FaMT1	gene10517	S-adenosyl-L-methionine-dependent methyltransferase	sense chain	AGGAGATAAGATAGCATTCGAAGTACCC	71,5	153	65	83,5	1,782±0,028	
			anti-sense chain	CTGTACTTAGGATCACAAGGCTTGAAC	70,9					
FaBZIP1	gene17796	Basic leucine zipper transcription factor	sense chain	AGGGTCAACAAAACCAGAATGGGGATAA	77,7	151	65	85,5	1,712±0,027	
			anti-sense chain	CTGCGTTCCAGCTCTGAAATGTATTGC	77,8					
FaTIM1	gene17570	Mitochondrial import inner membrane translocase	sense chain	GCTCCGCCACTTACGCCGCTAATTT	80,2	100	65	86,5	1,811±0,028	
			anti-sense chain	AGATCATCAGGCCCCGTCTTTCTCGTTA	80					
FaFHA1	gene17571	SMAD/FHA domain-containing protein	sense chain	ATTGCATGCTAAGTTGGTGGAACAGTAT	73,9	179	65	85,5	1,744±0,025	
			anti-sense chain	GACCCTTAGACCTTGTGTTGATGACAAA	74,6					
FaRIB413	gene33863	RNA interspacer (16S–23S) region	sense chain	ACCGTTGATTCGCACAATTGGTCATCG	83,4	149	65	91	1,784±0,032	[29]
			anti-sense chain	TACTGCGGGTCGGCAATCGGACG	81,9					
FaWRKY1	gene07210	WRKY75 like transcription factor	sense chain	ACAGCAGTAAGATTAGGGATGAAGAAGGGAG	76,2	196	65	85,5	1,824±0,025	[30]
			anti-sense chain	GCTTCTTCACATTGCAACCCTGATGCGTG	83,8					

(a) Accession number of genes found in *Fragaria vesca* genome (http://www.strawberrygenome.org/and http://www.rosaceae.org/) that may be amplified by the designed primer pairs. (b) Theoretical melting temperatures calculated by Oligo Primer Analisys software version 6.65 for each primer. (c) Recommended optimal annealing temperature was calculated by gradient PCR and subsequent PCR efficiency optimization. (d) PCR-product melting temperature as determined by melting curves (e) PCR efficiencies were calculated by LigRegPCR. (f) Known references for genes previously analyzed or described.

#### Primers designed of candidate reference genes

The RTqPCR primer pairs for each putative reference gene, as well as for *FaWRKY1*, were designed based on common criteria, and were tested to generate clear and unique PCR products in RTqPCR reactions (Table 1 and Figure S1).

All primers were designed from the CDS of the selected genes, avoiding regions of conserved sequence similarity to other genes. For genes belonging to gene families or with identified paralogs present in the genome of the diploid woodland strawberry (*F. vesca*) [35], the least conserved region was used to ensure amplification of a single gene by PCR. In four cases (*FaEF1α*, *FaTUBα*, *FaTUBβ* and *FaACTIN*), it was not possible to differentiate between either multicopy or nearly identical genes although unique amplicons were obtained. In six cases including the control gene (*FaGAPDH1*, *FaTUBβ*, *FaBZIP1*, *FaTIM1*, *FaFHA1*, *FaWRKY1*) primers were designed to span an exon-exon junction.

To ensure maximum specificity and efficiency during PCR amplification, primers were designed to have melting temperatures over 70 °C, and were required to generate short amplicons, usually between 100 and 200 bp (Table 1). The most appropriate annealing temperature for every primer pair was calculated by RTq-gradientPCR, and only primer pairs with optimal efficiency at annealing temperatures of above 65°C were considered for subsequent RTqPCR analyses. The primer pair for gene *FaRIB413* was previously designed in our group [29], and tested to meet all of the above criteria. The specificity of the primers was tested by PCR using first-strand cDNAs synthesized from total RNA isolated from the biological samples.

The PCR efficiency of each primer pair was calculated using LinRegPCR, a method that utilizes absolute fluorescence data captured during the exponential phase of amplification of each real-time PCR reaction [36]. Table 1 shows the calculated PCR efficiencies for the primer pairs studied. Each efficiency value represents an average ± SD calculated from 192 amplification plots (i.e. two technical replicates of two biological replicates of a total of 48 different experimental conditions). For all primer pairs, values ranged from 1.712 to 1.925, with low standard deviation. These values indicated comparable amplification efficiencies among the 96 diverse cDNA samples tested (Table 1), and suggested that the designed primer pairs efficiently amplified their target genes. Therefore, the mean primer pair efficiency value was considered for all subsequent studies, including estimations of the relative expression level of the reference genes under evaluation.

### Expression Stability of the Candidate Reference Genes under Different Experimental Conditions

Candidate reference genes were evaluated by RTqPCR analyses in response to the experimental conditions summarized in Table 2. Samples from different strawberry varieties were also examined. Two independent biological replicates were performed for each experimental condition. Between 10 and 18 independent samples per experiment were analyzed. In addition, two technical replicates corresponding to two biological replicates were used in this study. The generated results were subjected to the following analytical methods: analysis of ‘‘Stability index’’ [10], geNORM [4] implemented in qBASEplus software [37], NormFinder [9], BestKeeper [38], and the comparative Δ-Ct [39].

**Table 2:** Summary of strawberry varieties, tissues and experimental conditions used in this study.

Biological process	Cultivar	Culture type/Tissue	Biological stages/Time points after treatments	Experimental conditions	
Ripening and senescence	Camarosa	Fruit	G, W, R, OR and SE	Fruit ripening in field	RCF^a^
Defense against fungal infection	Camarosa	Fruit	Red stage fruits: Mock/Infectedgrades 1, 2, 3 and 4	Red fruit naturally infected with*C. acutatum* in field	FCF^a, e^
Defense against fungal infection	Camarosa	Crown	Mock: 1, 3, 5 and 7 dpi/Infected:1, 3, 5 and 7 dpi	Growth chamber *C. acutatum*infection under controlledconditions	FCC^b, e^
Defense against fungal infection	Camarosa	Petiole	Mock: 1, 3, 5 and 7 dpi/Infected:1, 3, 5 and 7 dpi	Growth chamber *C. acutatum*infection under controlledconditions	FCP^b, d, e^
Defense against fungal infection	Andana	Petiole	Mock: 3, 5 and 7 dpi/Infected:1, 3, 5 and 7 dpi	Growth chamber *C. acutatum*infection under controlledconditions	FAP^d^
Hormone response	Camarosa	Young in-vitro plant	Mock: 12, 24, 48hpt/SA (5 mM):12, 24, 48hpt/MeJA (2 mM):12, 24, 48hpt	Mock, SA and MeJA treatment	HCY^c, e^
Hormone response	Chandler	Cellular suspensions	Mock: 4 and 6hpt/SA (0,75 mM):4 and 6hpt/MeJA (0,1 mM):4 and 6hpt	Mock, SA and MeJA treatment	HCC^c^

RCF, Ripening-Camarosa-Fruit; FCF, Fungal-Camarosa-Fruit; FCC, Fungal-Camarosa-Crown; FCP, Fungal-Camarosa-Petiole; FAP, Fungal-Andana-Petiole; HCY, Hormone-Camarosa-Young-in-vitro; HCC, Hormone-Chandler-Cellular-suspensions. G1: small green, W: white, R: red, OR: over-ripened, SE: senescent.

(a) Comparison of gene expression between overripening-derived senescence and infection-derived necrosis, (b) Comparison of gene expression between cultivars under biotic stress, (c) Comparison of gene expression between cultivars under hormonal treatment, (d) Comparison of gene expression between different plant tissues, (e) Comparison of gene expression between infected and hormone treated plants.

#### Statistical analysis of gene expression by “stability index” calculation

**Figure 1:**
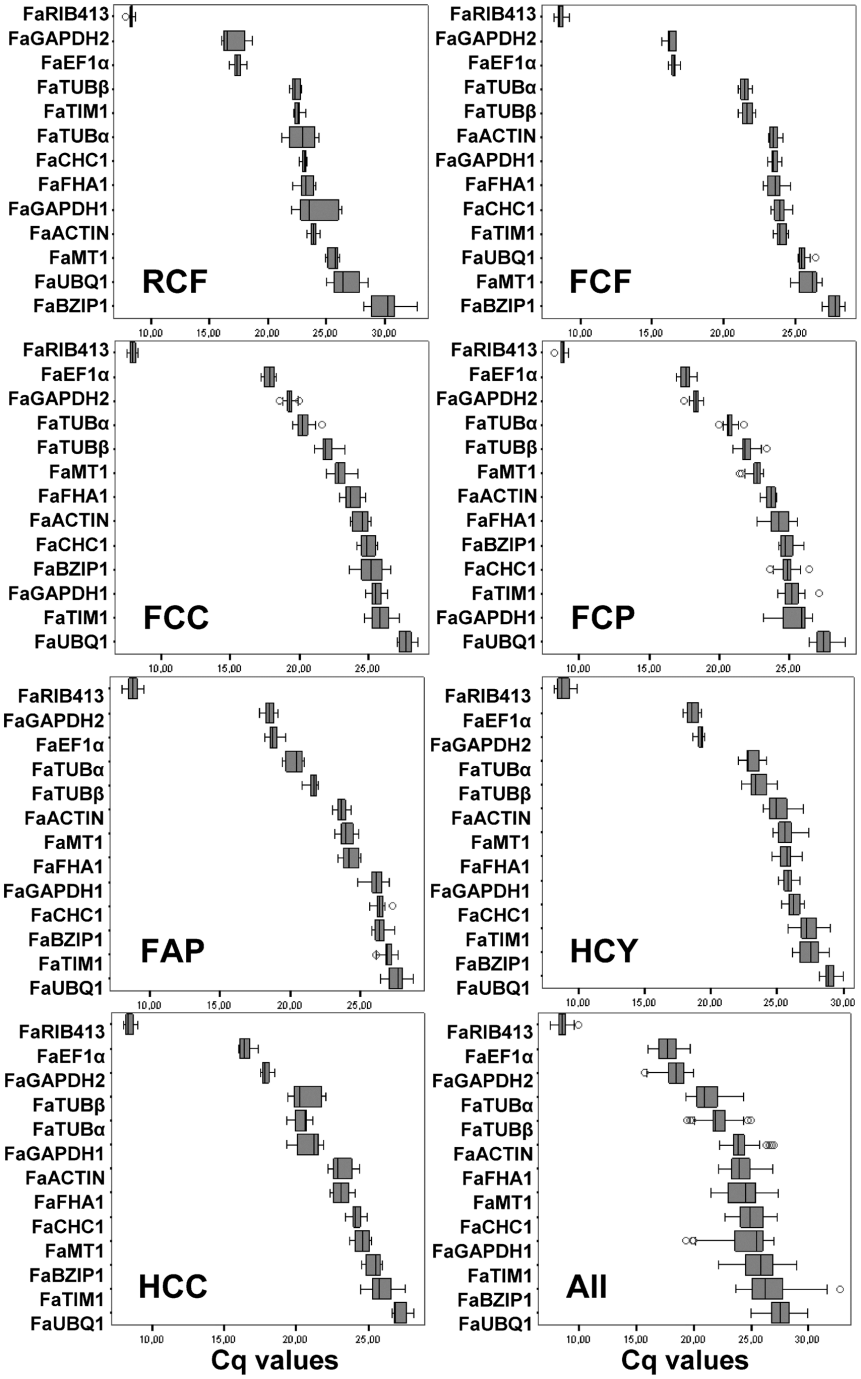
Expression levels of candidate reference genes in different experimental sets. Box plot graphs of Cq values for each reference gene tested in all strawberry samples and subsets. Cq values are inversely proportional to the amount of template and are shown as the first and third quartile. Vertical lines indicate the range of values, and median values are indicated by the black lines. Circles indicate outliers. RCF, Ripening-Camarosa-Fruit; FCF, Fungal-Camarosa-Fruit; FCC, Fungal-Camarosa-Crown; FCP, Fungal-Camarosa-Petiole; FAP, Fungal-Andana-Petiole; HCY, Hormone-Camarosa-Young-in-vitro; HCC, Hormone-Chandler-Cellular-suspensions; All, samples from all seven experiments analyzed together.

Figure 1 shows the expression level of candidate reference genes in the seven experimental conditions named in Table 2. Mean Cq values for each transcript in every experimental condition, together with coefficient of variation (CV), slope, and stability index (SI), according to Brunner, (2004) [10] are given in Table 3.

**Table 3:** Summary of statistics evaluating stability of gene expression.

		Mean ^b^	SD	CV (%)	Slope ^c^	Intercept	Stability index ^d^			Mean ^b^	SD	CV (%)	Slope ^c^	Intercept	Stability index ^d^
	**Ripening-Camarosa-Fruit (n = 20) ^a^**								**Fungal infection-Andana-Petiole (n = 28)**						
*	FaRIB413	8,341	0,239	2,860	0,004	8,329	0,011	*	FaGAPDH1	26,129	0,632	2,418	0,004	26,143	0,009
*	FaCHC1	23,085	0,201	0,869	0,021	23,024	0,018	*	FaGAPDH2	18,484	0,374	2,024	0,025	18,585	0,051
*	FaTUBβ	22,334	0,359	1,609	0,015	22,289	0,024	*	FaACTIN	23,640	0,428	1,812	0,030	23,760	0,054
	FaACTIN	23,894	0,309	1,294	0,144	24,326	0,186	*	FaEF1α	18,780	0,417	2,219	0,026	18,886	0,059
	FaTIM1	22,602	0,359	1,587	0,151	23,054	0,239	*	FaMT1	23,992	0,506	2,108	0,028	23,879	0,060
	FaMT1	25,622	0,449	1,753	0,143	25,193	0,251	*	FaFHA1	24,251	0,570	2,349	0,036	24,107	0,085
	FaEF1α	17,406	0,413	2,371	0,161	17,889	0,382		FaTUBβ	21,575	0,374	1,734	0,090	21,216	0,155
	FaFHA1	23,258	0,643	2,765	0,204	23,870	0,564		FaCHC1	26,339	0,404	1,536	0,120	26,816	0,184
	FaTUBα	22,899	1,174	5,128	0,556	24,567	2,851		FaBZIP1	26,410	0,446	1,688	0,110	25,971	0,185
	FaBZIP1	30,089	1,485	4,936	0,607	28,270	2,994		FaTIM1	26,864	0,481	1,790	0,116	26,401	0,207
	FaUBQ1	26,677	1,249	4,680	0,812	29,113	3,800		FaUBQ1	27,650	0,642	2,322	0,115	27,085	0,266
	FaGAPDH2	17,073	1,071	6,274	0,622	18,939	3,903		FaRIB413	8,790	0,444	5,053	0,061	9,036	0,310
	FaGAPDH1	24,080	1,715	7,120	1,115	27,425	7,939		FaTUBα	20,281	0,595	2,931	0,211	19,436	0,620
	**Fungal infection-Camarosa-Fruit (n = 20)**								**Hormonal treatment-Camarosa-Young in-vitro plant (n = 36)**						
*	FaGAPDH1	23,530	0,316	1,345	0,005	23,545	0,007	*	FaGAPDH1	25,817	0,479	1,856	0,024	25,938	0,045
*	FaTUBα	21,462	0,322	1,499	0,019	21,518	0,028	*	FaUBQ1	28,954	0,518	1,789	0,026	29,085	0,047
*	FaUBQ1	25,599	0,405	1,583	0,047	25,458	0,074	*	FaGAPDH2	19,183	0,278	1,451	0,038	18,993	0,055
	FaGAPDH2	16,274	0,331	2,031	0,062	16,090	0,125	*	FaRIB413	8,838	0,523	5,912	0,016	8,760	0,093
	FaACTIN	23,539	0,314	1,335	0,136	23,133	0,181		FaCHC1	26,297	0,482	1,832	0,080	25,895	0,147
	FaEF1α	16,556	0,250	1,510	0,130	16,166	0,196		FaTUBα	23,058	0,586	2,542	0,093	22,591	0,237
	FaTIM1	24,031	0,372	1,549	0,131	23,638	0,203		FaFHA1	25,649	0,615	2,396	0,101	25,145	0,242
	FaCHC1	23,929	0,467	1,953	0,121	23,568	0,235		FaEF1α	18,593	0,442	2,375	0,119	17,996	0,284
	FaTUBβ	21,668	0,387	1,784	0,133	21,271	0,236		FaMT1	25,669	0,726	2,829	0,165	24,846	0,466
	FaBZIP1	27,780	0,478	1,719	0,164	27,288	0,282		FaTIM1	27,336	0,800	2,928	0,176	26,457	0,514
	FaFHA1	23,606	0,545	2,308	0,213	22,969	0,490		FaTUBβ	23,573	0,775	3,286	0,218	22,484	0,716
	FaRIB413	8,635	0,323	3,736	0,158	8,161	0,590		FaBZIP1	27,459	0,845	3,079	0,262	26,150	0,806
	FaMT1	25,910	0,745	2,876	0,425	24,635	1,222		FaACTIN	25,122	0,979	3,899	0,325	23,499	1,265
	**Fungal infection-Camarosa-Crown (n = 32)**								**Hormonal treatment-Chandler-Cellular suspensions (n = 24)**						
*	FaUBQ1	27,734	0,486	1,752	0,037	27,567	0,065	*	FaTIM1	25,850	0,974	3,766	0,003	25,862	0,013
*	FaRIB413	7,873	0,241	3,057	0,027	7,752	0,083	*	FaGAPDH2	17,889	0,300	1,679	0,013	17,843	0,022
	FaGAPDH1	25,569	0,453	1,771	0,064	25,282	0,113	*	FaRIB413	8,426	0,299	3,551	0,021	8,498	0,073
	FaCHC1	24,988	0,492	1,968	0,067	24,687	0,131	*	FaUBQ1	27,222	0,566	2,079	0,039	27,322	0,081
	FaEF1α	17,786	0,386	2,173	0,062	17,509	0,134		FaCHC1	24,163	0,427	1,767	0,129	23,712	0,228
	FaGAPDH2	19,286	0,352	1,825	0,090	18,880	0,165		FaBZIP1	25,344	0,523	2,063	0,163	25,914	0,336
	FaMT1	22,968	0,571	2,486	0,068	22,664	0,168		FaEF1α	16,478	0,413	2,505	0,151	15,950	0,377
	FaFHA1	23,875	0,651	2,728	0,062	24,156	0,170		FaTUBα	20,364	0,539	2,649	0,236	19,538	0,625
	FaTIM1	25,885	0,813	3,139	0,116	25,363	0,364		FaMT1	24,533	0,550	2,240	0,282	23,545	0,632
	FaTUBβ	22,086	0,622	2,818	0,136	21,472	0,384		FaFHA1	23,116	0,612	2,646	0,246	22,256	0,650
	FaTUBα	20,298	0,585	2,883	0,136	19,687	0,392		FaACTIN	23,145	0,776	3,352	0,343	21,943	1,151
	FaACTIN	24,440	0,563	2,303	0,211	23,493	0,485		FaGAPDH1	20,908	0,836	3,999	0,401	22,313	1,605
	FaBZIP1	25,229	0,929	3,682	0,279	23,975	1,026		FaTUBβ	20,592	0,964	4,681	0,436	19,067	2,040
	**Fungal infection-Camarosa-Petiole (n = 32)**								**All seven experiments (n = 192)**						
*	FaTUBα	20,767	0,423	2,036	0,007	20,737	0,014	*	FaACTIN	24,011	0,883	3,676	0,004	23,905	0,015
*	FaACTIN	23,676	0,364	1,536	0,013	23,528	0,020		FaRIB413	8,542	0,490	5,736	0,056	8,306	0,323
*	FaRIB413	8,816	0,237	2,685	0,027	8,695	0,072		FaTUBβ	22,073	1,067	4,835	0,069	22,252	0,333
*	FaBZIP1	24,874	0,620	2,493	0,034	25,025	0,084		FaEF1α	17,716	0,904	5,100	0,082	17,270	0,416
*	FaEF1α	17,574	0,437	2,485	0,034	17,727	0,084		FaMT1	24,338	1,399	5,747	0,097	24,857	0,560
	FaGAPDH2	18,321	0,357	1,946	0,064	18,611	0,125		FaFHA1	24,140	1,037	4,298	0,144	23,426	0,619
	FaMT1	22,583	0,517	2,288	0,065	22,293	0,148		FaTUBα	21,292	1,305	6,131	0,158	21,937	0,970
	FaTUBβ	22,009	0,574	2,609	0,100	21,561	0,260		FaGAPDH1	24,722	1,856	7,509	0,157	25,119	1,175
	FaCHC1	24,874	0,735	2,954	0,139	25,499	0,411		FaUBQ1	27,492	1,134	4,124	0,295	26,149	1,217
	FaUBQ1	27,470	0,766	2,788	0,169	28,246	0,472		FaGAPDH2	18,270	1,063	5,818	0,267	17,007	1,551
	FaTIM1	25,223	0,755	2,993	0,193	26,091	0,577		FaCHC1	25,000	1,189	4,756	0,333	23,479	1,583
	FaFHA1	24,264	0,774	3,191	0,224	25,270	0,713		FaBZIP1	26,547	1,789	6,741	0,489	28,697	3,297
	FaGAPDH1	25,419	1,120	4,405	0,263	26,600	1,156		FaTIM1	25,650	1,591	6,201	0,619	22,923	3,839

Genes are ordered into each experiment analyzed, top to bottom, from those tending to show the highest stability to those showing the lowest, based on the stability index. a) "n" represents the number of individuals analyzed from each experiment, (four data points per sample, two biological and two technical replicates of each). b) Data based on analysis of Cq values. SD, standard deviation. CV, Coeficient of variation. c) Slope of regression of gene means. Intercepts are also given for the estimated regression lines. d) Stability index is the product of CV and slope (multiplication of columns 3 and 4). Transcripts with lower slope are preferred as controls. Asterisks mark the best candidate genes with stability index below 0.0×.

The analysis of variation, as reflected in the coefficient of variation (CV), showed highly predictability of all candidate reference genes in every of the seven experimental conditions. Considering them together, almost all CV values were below 6%. Exceptions were genes *FaGAPDH1* and *FaGAPDH2*, which deviated substantially during ripening, and genes *FaTUBα, FaGAPDH1, FaBZIP1* and *FaTIM1*, within the “all together” conditions (Table 3).

The mean expression level for each gene was regressed against the overall means for the different samples (Figure S2). The slope of the predicted regression lines provided an estimate of the degree to which the gene is sensitive to general expression-promoting conditions. Assuming that both consistent transcript levels among samples (low slope) and high predictability (low CV) are desired, the “stability index” (SI) (product of slope and CV) is used to describe transcript stability as in Brunner, (2004) [10]. Transcripts with the lowest stability index will usually provide the best reference genes or controls.

The results show that several predicted candidate genes show a favorable SI in each of the main areas studied (Table 3, marked by asterisks). During fruit ripening candidates *FaRIB413, FaCHC1* and *FaTUBβ* showed low SI values (0.011, 0.018, and 0.024, respectively). Genes *FaGAPDH1*, *FaTUBα* and *FaUBQ1* also appear to be excellent reference genes for fungal infection studies in red fruit (SI of 0.007, 0.028, and 0.074, respectively). In vegetative tissues challenged with the fungus, variations in number and diversity of convenient reference genes was also found. Thus, genes *FaUBQ1* (SI, 0.065) and *FaRIB413* (SI, 0.083), were found to be the best candidates for normalization on crown tissue of cultivar Camarosa but genes *FaTUBα* (SI, 0.014), *FaACTIN* (SI, 0.020), *FaRIB413* (SI, 0.072), *FaBZIP1* (SI, 0.084), *FaEF1α* (SI, 0.084) were also very good candidates on petiole tissue of this cultivar. However, on petiole tissue from cultivar Andana, the set of predicted good candidate reference genes is not the same. The best candidates were genes *FaGAPDH1* (SI, 0.009), *FaGAPDH2* (SI, 0.051), *FaACTIN* (SI, 0.054), *FaEF1α* (SI, 0.059), *FaMT1* (SI, 0.060), and *FaFHA1* (SI, 0.085). Only genes *FaACTIN* and *FaEF1α* were found to be the reasonable reference genes for normalization in petiole tissue of both strawberry cultivars. In addition, genes *FaUBQ1*, *FaGAPDH2*, and *FaRIB413* were found to be the optimal reference genes for SA and JA studies either in in-vitro plants (SI, 0.047, 0.055, and 0.093, respectively) in cell suspension treatments (SI, 0.081, 0.022, and 0.073, respectively), as well as across different cultivars. Genes *FaGAPDH1* (SI, 0.045) and *FaTIM1* (SI, 0.013) were also found to be appropriate candidates for the in-vitro plants and cellular suspension experiments, respectively.

Also, we have considered an “all together” analysis where all seven experimental variables have been examined. In this analysis, gene *FaACTIN* showed the lowest stability index (SI, 0.015), and appears to be the best overall reference gene following this analytical method.

#### Expression stability and calculation of hypothetical normalization factor by geNorm^PLUS^

The stability coeficient (M values) and the coefficient of variation (CV values) of each gene are inversely related to their expression stability. These values were calculated using qBase software [37] but taking into account the previously calculated specific PCR efficiency of each gene. The average stability coefficient (M_A_), defined as the average value of the M values (average pairwise variation of a gene with all other tested reference genes of all combinations of a gene and high-ranking reference genes) of the relative quantities of the thirteen genes under evaluation were analyzed with geNormPlus (qBase software, [4], [37]).

**Figure 2:**
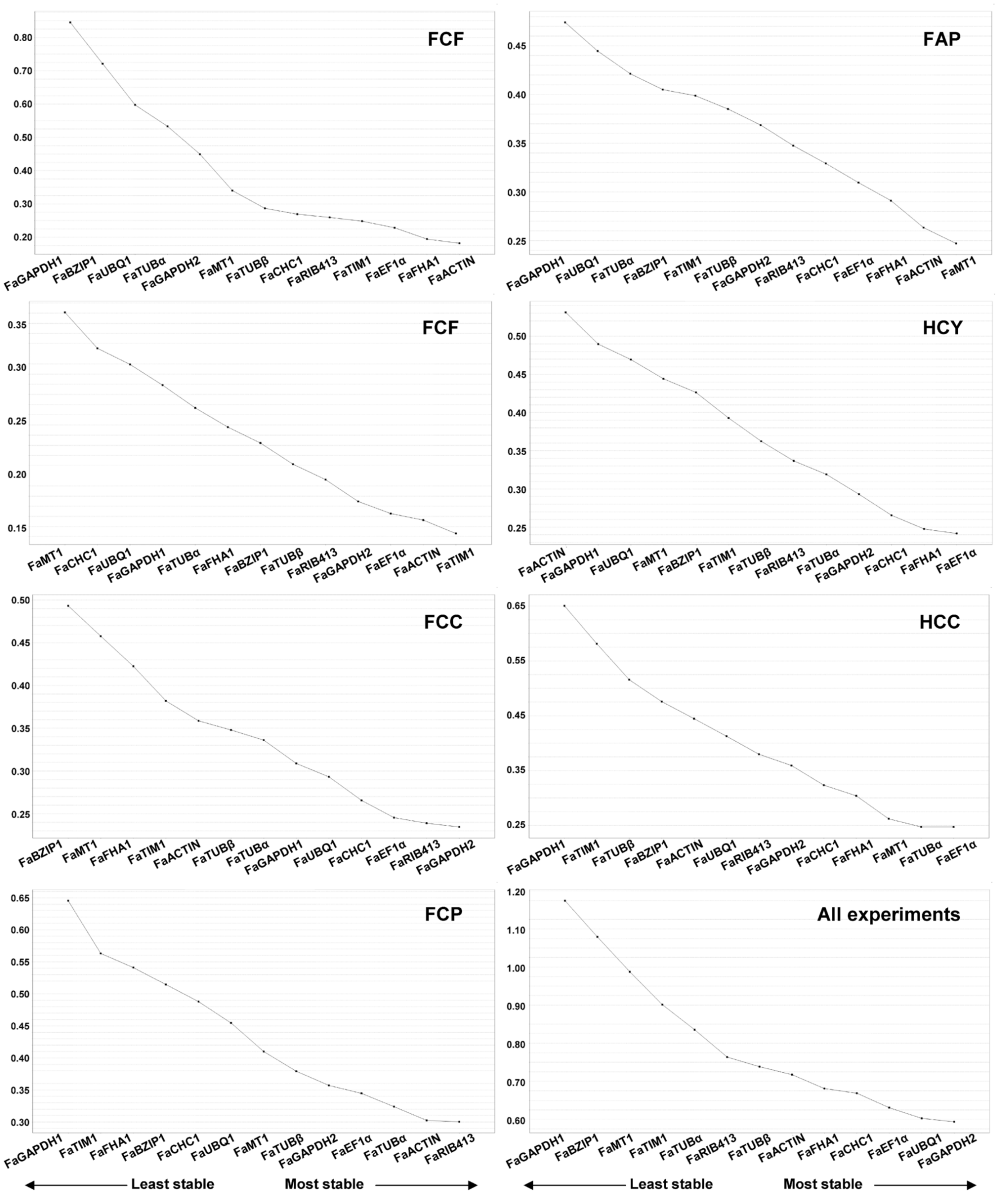
Average expression stability value (M_A_) of each gene. Specific M_A_ values were calculated under seven single experimental conditions tested, as well as by combining all samples together. M_A_ for genes tested are shown as derived by geNormPLUS analysis. The lowest M_A_ value indicates the most stable expression.

Figure 2 represents the average stability coefficients (M_A_) of the thirteen candidate reference genes tested from every analyzed condition. All thirteen genes showed acceptable expression stabilities (M_A_≤1), as described for heterogeneous samples [37], with the exception of genes *FaBZIP1* and *FaGAPDH1* when all seven experimental conditions were analyzed together.

Table 4 shows transcripts ranked by their M_A_ and CV values. The M_A_ results revealed that optimal candidate reference genes differed among the analyzed experimental conditions. Thus, *FaACTIN* (0.182) seems to be the most stable gene in fruit ripening analyses, meanwhile *FaTIM1* (0.143) is in fruit natural infection, *FaGAPDH2* (0.234) and FaRIB413 (0.300) in Camarosa crown and petiole infected tissues, respectively, *FaMT1* (0.247) in Andana infected petiole, *FaEF1α* (0.242) in hormonal treatments of in-vitro plants, *FaEF1α* (0.242) and *FaTUBα* (0.242) in elicited cellular suspensions of cultivar Chandler, and finally, *FaGAPDH2* (0.594) in the “all together” conditions. A similar result was obtained when CV values were considered.

**Table 4:** Reference genes ranked in order by their average expression stability (MA) and coefficient of variation (CV) respectively.

Ranking by M_A_ values from geNorm^PLUS^													
**RCF**	FaGAPDH1	FaBZIP1	FaUBQ1	FaTUBα	FaGAPDH2	FaMT1	FaTUBβ	FaCHC1	FaRIB413	FaTIM1	FaEF1α	FaFHA1	FaACTIN
	(0.845)	(0.712)	(0.597)	(0.533)	(0.449)	(0.34)	(0.287)	(0.269)	(0.259)	(0.248)	(0.228)	(0.195)	(0.182)
**FCF**	FaMT1	FaCHC1	FaUBQ1	FaGAPDH1	FaTUBα	FaFHA1	FaBZIP1	FaTUBβ	FaRIB413	FaGAPDH2	FaEF1α	FaACTIN	FaTIM1
	(0.361)	(0.325)	(0.31)	(0.289)	(0.267)	(0.248)	(0.232)	(0.211)	(0.196)	(0.175)	(0.163)	(0.156)	(0.143)
**FCC**	FaBZIP1	FaMT1	FaFHA1	FaTIM1	FaACTIN	FaTUBβ	FaTUBα	FaGAPDH1	FaUBQ1	FaCHC1	FaEF1α	FaRIB413	FaGAPDH2
	(0.493)	(0.458)	(0.422)	(0.382)	(0.359)	(0.348)	(0.336)	(0.309)	(0.293)	(0.266)	(0.245)	(0.239)	(0.234)
**FCP**	FaGAPDH1	FaTIM1	FaFHA1	FaBZIP1	FaCHC1	FaUBQ1	FaMT1	FaTUBβ	FaGAPDH2	FaEF1α	FaTUBα	FaACTIN	FaRIB413
	(0.645)	(0.563)	(0.541)	(0.515)	(0.488)	(0.454)	(0.41)	(0.379)	(0.357)	(0.345)	(0.324)	(0.302)	(0.3)
**FAP**	FaGAPDH1	FaUBQ1	FaTUBα	FaBZIP1	FaTIM1	FaTUBβ	FaGAPDH2	FaRIB413	FaCHC1	FaEF1α	FaFHA1	FaACTIN	FaMT1
	(0.474)	(0.445)	(0.421)	(0.405)	(0.399)	(0.385)	(0.369)	(0.348)	(0.329)	(0.31)	(0.291)	(0.263)	(0.247)
**HCY**	FaACTIN	FaGAPDH1	FaUBQ1	FaMT1	FaBZIP1	FaTIM1	FaTUBβ	FaRIB413	FaTUBα	FaGAPDH2	FaCHC1	FaFHA1	FaEF1α
	(0.531)	(0.49)	(0.469)	(0.444)	(0.426)	(0.393)	(0.362)	(0.337)	(0.319)	(0.293)	(0.266)	(0.248)	(0.242)
**HCC**	FaGAPDH1	FaTIM1	FaTUBβ	FaBZIP1	FaACTIN	FaUBQ1	FaRIB413	FaGAPDH2	FaCHC1	FaFHA1	FaMT1	FaTUBα	FaEF1α
	(0.651)	(0.581)	(0.516)	(0.476)	(0.445)	(0.413)	(0.38)	(0.359)	(0.323)	(0.304)	(0.262)	(0.247)	(0.247)
**All samples**	FaGAPDH1	FaBZIP1	FaMT1	FaTIM1	FaTUBα	FaRIB413	FaTUBβ	FaACTIN	FaFHA1	FaCHC1	FaEF1α	FaUBQ1	FaGAPDH2
	(1.174)	(1.079)	(0.987)	(0.901)	(0.835)	(0.764)	(0.738)	(0.717)	(0.681)	(0.669)	(0.631)	(0.603)	(0.594)
**Ranking by CV values from geNorm^PLUS^**													
**RCF**	FaGAPDH1	FaBZIP1	FaUBQ1	FaMT1	FaTUBα	FaGAPDH2	FaTUBβ	FaCHC1	FaRIB413	FaTIM1	FaFHA1	FaACTIN	FaEF1α
	(0.844)	(0.703)	(0.493)	(0.487)	(0.441)	(0.337)	(0.299)	(0.292)	(0.234)	(0.17)	(0.17)	(0.155)	(0.093)
**FCF**	FaMT1	FaGAPDH1	FaTUBα	FaCHC1	FaUBQ1	FaFHA1	FaTIM1	FaACTIN	FaBZIP1	FaTUBβ	FaRIB413	FaGAPDH2	FaEF1α
	(0.352)	(0.236)	(0.219)	(0.206)	(0.188)	(0.171)	(0.144)	(0.139)	(0.118)	(0.116)	(0.116)	(0.1)	(0.073)
**FCC**	FaBZIP1	FaMT1	FaFHA1	FaTIM1	FaUBQ1	FaCHC1	FaACTIN	FaGAPDH1	FaTUBβ	FaGAPDH2	FaTUBα	FaEF1α	FaRIB413
	(0.374)	(0.369)	(0.311)	(0.278)	(0.244)	(0.204)	(0.204)	(0.201)	(0.164)	(0.147)	(0.145)	(0.136)	(0.111)
**FCP**	FaGAPDH1	FaMT1	FaFHA1	FaCHC1	FaTIM1	FaTUBβ	FaBZIP1	FaACTIN	FaUBQ1	FaTUBα	FaEF1α	FaRIB413	FaGAPDH2
	(0.758)	(0.372)	(0.325)	(0.308)	(0.304)	(0.297)	(0.257)	(0.246)	(0.237)	(0.205)	(0.191)	(0.166)	(0.136)
**FAP**	FaGAPDH1	FaUBQ1	FaTUBα	FaRIB413	FaACTIN	FaCHC1	FaGAPDH2	FaMT1	FaBZIP1	FaTIM1	FaTUBβ	FaFHA1	FaEF1α
	(0.393)	(0.319)	(0.266)	(0.24)	(0.239)	(0.215)	(0.206)	(0.205)	(0.201)	(0.183)	(0.175)	(0.154)	(0.12)
**HCY**	FaACTIN	FaGAPDH1	FaTIM1	FaUBQ1	FaMT1	FaBZIP1	FaRIB413	FaTUBβ	FaTUBα	FaGAPDH2	FaFHA1	FaCHC1	FaEF1α
	(0.375)	(0.373)	(0.34)	(0.295)	(0.282)	(0.277)	(0.224)	(0.203)	(0.181)	(0.18)	(0.173)	(0.15)	(0.122)
**HCC**	FaGAPDH1	FaTIM1	FaTUBβ	FaACTIN	FaBZIP1	FaMT1	FaGAPDH2	FaUBQ1	FaFHA1	FaRIB413	FaTUBα	FaEF1α	FaCHC1
	(0.685)	(0.47)	(0.406)	(0.314)	(0.282)	(0.254)	(0.229)	(0.223)	(0.209)	(0.2)	(0.176)	(0.159)	(0.155)
**All samples**	FaGAPDH1	FaTIM1	FaMT1	FaBZIP1	FaCHC1	FaTUBα	FaGAPDH2	FaUBQ1	FaRIB413	FaTUBβ	FaACTIN	FaFHA1	FaEF1α
	(1.703)	(1.072)	(0.721)	(0.595)	(0.581)	(0.533)	(0.521)	(0.474)	(0.419)	(0.376)	(0.332)	(0.279)	(0.259)

Increasing stability from left to right. See Table 2 for experimental description.

**Figure 3:**
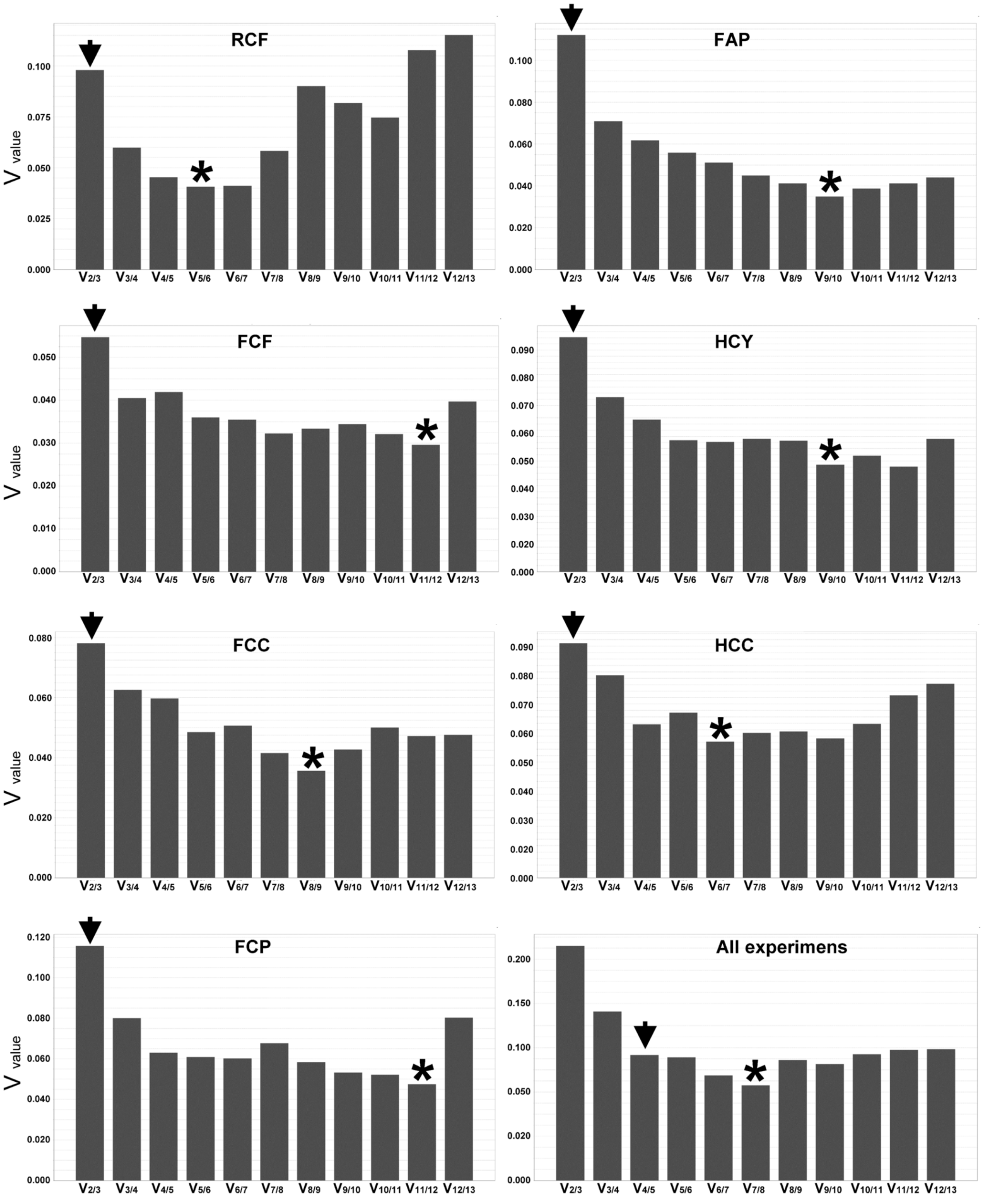
Determination of the number of genes required to calculate a hypothetical normalization factor. Pairwise variation (Vn/n+1) analysis was carried out to determine the number of reference genes required for accurate normalization. An asterisk indicates the lowest V value in each experiment. An arrowhead indicates the minimum number of genes required to pass the suggested cut-off value (0.15) [4]. See Table 2 for experiment description.

The optimal and the minimal number of reference genes needed to calculate a hypothetical optimal normalization factor suitable in each analyzed condition was determined, as described by Vandesompele [4]. Figure 3, shows that the optimal number (V) of these needed reference genes differed in each experimental conditions but a combination of them is assumed to be an ideal reference gene. Thus, in fruit ripening analyses, V_5/6_ was the lowest pairwise variation value (0.041). Therefore, the hypothetical normalization factor in these experimental conditions would be the geometric mean of the five or six more stable genes (see Figure 2 and Table 4, for the ranking of more stable genes for this and other experimental condition). Other lowest pairwise variation values were, V_11/12_ (0.03) for the infected fruit experiment, V_8/9_ (0.036) and V_11/12_ (0.047) for Camarosa crown and petiole infected tissues, respectively, V_9/10_ (0.035) for Andana infected petioles, V_9/10_ (0.043) for hormonal treatment of in-vitro plants experiment, V_6/7_ (0.053) for elicited cellular suspensions, and finally, V_7/8_ (0.086) when all experiments were considered together.

In practice, however, the number of genes required should be low enough to make experimental procedures affordable, yet high enough to merit confidence in the conclusions. This means that if the pairwise variation value for n genes is below the recommended cut-off of 0.15, additional genes will not likely contribute to improved normalization [4]. Thus, the minimal number of reference candidates in each single experiment was determined to be two, for all the experimental conditions tested (marked with an arrowhead in Figure 3), but four in the all-together conditions. In each experimental condition, these genes were *FaACTIN* and *FaFHA1* (V_2/3_ value of 0.098) for fruit ripening, *FaTIM1* and *FaACTIN* (V_2/3_ value of 0.055) for fruit infection, *FaGAPDH2* and *FaRIB413* (V_2/3_ value of 0.078) for Camarosa crown infection, *FaRIB413* and *FaACTIN* (V_2/3_ value of 0.116) for Camarosa petiole infection, *FaMT1* and *FaACTIN* (V_2/3_ value of 0.112) for Andana petiole infection, *FaEF1α* and *FaFHA1* (V_2/3_ value of 0.095) for in-vitro plants treated with hormones, and *FaEF1α* and *FaTUBα* (V_2/3_ value of 0.091) for elicited cellular suspensions. For the all-together conditions the minimal reference genes were *FaGAPDH2*, *FaUBQ1*, *FaEF1α*, and *FaCHC1* (V_4/5_ value of 0.113).

#### Evaluation of expression stability by ΔCt method, Normfinder and BestKeeper approaches

In order to accurately assess the usefulness of the thirteen candidate reference genes, other analytical methods were applied to the same data set. These include the comparative ΔCt method [39], which ranks the reference genes by their mean standard deviation in the pairwise comparisons. The NormFinder [9] method was also used. NormFinder ranks the set of candidate normalization genes according to their expression stability in a given sample set and a given experimental design. The Bestkeeper algorithm [38] performs pairwise comparison using the geometric mean of the Cp (Cq), values, and this one was also implemented.

Table 5 shows the results obtained from all three methods. Both ΔCt and NormFinder analyses indicated a similar set of ideal reference genes for each experimental condition. Essentially, the best were *FaTIM1* for ripening, *FaEF1α* for infected fruits, *FaEF1α* and *FaGAPDH2* for Camarosa crown and petiole infected tissues, respectively, *FaACTIN* for Andana infected petioles, *FaRIB413* for in-vitro hormone-treated plants, *FaRIB413* for cellular suspension treatments, and finally, *FaEF1α* when all the experiments were analyzed together. Similar results were also obtained when BestKeeper algorithm was used.

**Table 5:** Ranking of candidate reference genes based on expression stability as assessed by ΔCt, Normfinder and BestKeeper methods.

Ranking by STDEV values from ΔCt													
**RCF**	FaBZIP1	FaGAPDH1	FaUBQ1	FaTUBα	FaCHC1	FaMT1	FaFHA1	FaGAPDH2	FaTUBβ	FaEF1α	FaRIB413	FaACTIN	FaTIM1
	(1.64)	(1.60)	(1.29)	(1.15)	(1.03)	(0.99)	(0.99)	(0.96)	(0.93)	(0.85)	(0.84)	(0.82)	(0.82)
**FCF**	FaBZIP1	FaMT1	FaFHA1	FaTIM1	FaCHC1	FaUBQ1	FaGAPDH2	FaTUBβ	FaTUBα	FaRIB413	FaGAPDH1	FaACTIN	FaEF1α
	(0.72)	(0.69)	(0.65)	(0.62)	(0.62)	(0.61)	(0.59)	(0.57)	(0.57)	(0.54)	(0.54)	(0.50)	(0.47)
**FCC**	FaBZIP1	FaFHA1	FaGAPDH1	FaTIM1	FaTUBβ	FaCHC1	FaACTIN	FaRIB413	FaMT1	FaGAPDH2	FaTUBα	FaUBQ1	FaEF1α
	(1.12)	(1.08)	(1.07)	(1.04)	(0.99)	(0.96)	(0.96)	(0.95)	(0.94)	(0.88)	(0.88)	(0.88)	(0.82)
**FCP**	FaGAPDH1	FaFHA1	FaTIM1	FaBZIP1	FaTUBβ	FaCHC1	FaMT1	FaACTIN	FaUBQ1	FaEF1α	FaTUBα	FaRIB413	FaGAPDH2
	(1.28)	(1.11)	(1.07)	(1.04)	(1.02)	(1.00)	(0.96)	(0.90)	(0.89)	(0.89)	(0.87)	(0.83)	(0.76)
**FAP**	FaGAPDH1	FaFHA1	FaUBQ1	FaBZIP1	FaTIM1	FaRIB413	FaGAPDH2	FaCHC1	FaMT1	FaTUBα	FaEF1α	FaTUBβ	FaACTIN
	(0.94)	(0.82)	(0.80)	(0.75)	(0.74)	(0.74)	(0.72)	(0.70)	(0.68)	(0.68)	(0.60)	(0.59)	(0.59)
**HCY**	FaACTIN	FaTIM1	FaBZIP1	FaGAPDH1	FaTUBβ	FaMT1	FaUBQ1	FaFHA1	FaCHC1	FaTUBα	FaEF1α	FaGAPDH2	FaRIB413
	(0.89)	(0.85)	(0.83)	(0.83)	(0.82)	(0.82)	(0.80)	(0.80)	(0.73)	(0.71)	(0.71)	(0.70)	(0.68)
**HCC**	FaGAPDH1	FaTIM1	FaTUBβ	FaACTIN	FaUBQ1	FaMT1	FaGAPDH2	FaBZIP1	FaFHA1	FaTUBα	FaCHC1	FaEF1α	FaRIB413
	(1.14)	(1.13)	(1.06)	(0.92)	(0.89)	(0.84)	(0.81)	(0.79)	(0.78)	(0.75)	(0.72)	(0.70)	(0.70)
**All samples**	FaBZIP1	FaGAPDH1	FaMT1	FaTIM1	FaTUBα	FaCHC1	FaGAPDH2	FaUBQ1	FaTUBβ	FaRIB413	FaFHA1	FaACTIN	FaEF1α
	(2.02)	(1.90)	(1.79)	(1.70)	(1.50)	(1.39)	(1.37)	(1.34)	(1.34)	(1.32)	(1.28)	(1.24)	(1.21)
**Ranking by stability values from NormFinder**													
**RCF**	FaBZIP1	FaGAPDH1	FaUBQ1	FaTUBα	FaCHC1	FaMT1	FaFHA1	FaTUBβ	FaGAPDH2	FaRIB413	FaEF1α	FaACTIN	FaTIM1
	(1.533)	(1.498)	(1.103)	(0.845)	(0.738)	(0.638)	(0.638)	(0.571)	(0.535)	(0.396)	(0.379)	(0.267)	(0.243)
**FCF**	FaBZIP1	FaMT1	FaFHA1	FaTIM1	FaCHC1	FaUBQ1	FaGAPDH2	FaTUBα	FaTUBβ	FaGAPDH1	FaRIB413	FaACTIN	FaEF1α
	(0.610)	(0.565)	(0.523)	(0.466)	(0.466)	(0.444)	(0.430)	(0.397)	(0.387)	(0.343)	(0.341)	(0.277)	(0.177)
**FCC**	FaBZIP1	FaGAPDH1	FaFHA1	FaTIM1	FaTUBβ	FaACTIN	FaCHC1	FaRIB413	FaMT1	FaUBQ1	FaGAPDH2	FaTUBα	FaEF1α
	(0.907)	(0.856)	(0.840)	(0.784)	(0.745)	(0.673)	(0.670)	(0.662)	(0.630)	(0.573)	(0.571)	(0.554)	(0.429)
**FCP**	FaGAPDH1	FaFHA1	FaTIM1	FaTUBβ	FaBZIP1	FaCHC1	FaMT1	FaACTIN	FaEF1α	FaUBQ1	FaTUBα	FaRIB413	FaGAPDH2
	(1.119)	(0.890)	(0.821)	(0.807)	(0.800)	(0.723)	(0.673)	(0.605)	(0.559)	(0.552)	(0.543)	(0.429)	(0.272)
**FAP**	FaGAPDH1	FaFHA1	FaUBQ1	FaBZIP1	FaTIM1	FaRIB413	FaGAPDH2	FaCHC1	FaTUBα	FaMT1	FaEF1α	FaTUBβ	FaACTIN
	(0.809)	(0.661)	(0.637)	(0.564)	(0.550)	(0.548)	(0.507)	(0.478)	(0.463)	(0.439)	(0.300)	(0.283)	(0.277)
**HCY**	FaACTIN	FaTIM1	FaGAPDH1	FaBZIP1	FaMT1	FaUBQ1	FaTUBβ	FaFHA1	FaCHC1	FaTUBα	FaEF1α	FaGAPDH2	FaRIB413
	(0.726)	(0.643)	(0.639)	(0.633)	(0.616)	(0.614)	(0.600)	(0.581)	(0.461)	(0.460)	(0.432)	(0.425)	(0.413)
**HCC**	FaGAPDH1	FaTIM1	FaTUBβ	FaACTIN	FaUBQ1	FaMT1	FaGAPDH2	FaBZIP1	FaFHA1	FaTUBα	FaCHC1	FaEF1α	FaRIB413
	(1.013)	(0.972)	(0.932)	(0.746)	(0.647)	(0.604)	(0.525)	(0.490)	(0.473)	(0.401)	(0.356)	(0.324)	(0.297)
**All samples**	FaBZIP1	FaGAPDH1	FaMT1	FaTIM1	FaTUBα	FaCHC1	FaGAPDH2	FaUBQ1	FaTUBβ	FaRIB413	FaFHA1	FaACTIN	FaEF1α
	(1.795)	(1.626)	(1.493)	(1.397)	(1.075)	(0.932)	(0.918)	(0.840)	(0.787)	(0.734)	(0.686)	(0.578)	(0.538)
**Ranking by SD of Cp from BestKeeper**													
**RCF**	FaGAPDH1	FaBZIP1	FaTIM1	FaMT1	FaTUBα	FaCHC1	FaTUBβ	FaUBQ1	FaFHA1	FaGAPDH2	FaEF1α	FaACTIN	FaRIB413
	(1.52)	(1.36)	(1.34)	(1.26)	(1.09)	(1.06)	(0.95)	(0.89)	(0.88)	(0.85)	(0.82)	(0.76)	(0.35)
**FCF**	FaMT1	FaBZIP1	FaCHC1	FaTIM1	FaTUBβ	FaGAPDH2	FaUBQ1	FaRIB413	FaFHA1	FaGAPDH1	FaACTIN	FaTUBα	FaEF1α
	(0.60)	(0.56)	(0.48)	(0.48)	(0.42)	(0.32)	(0.32)	(0.32)	(0.20)	(0.18)	(0.18)	(0.18)	(0.00)
**FCC**	FaBZIP1	FaTUBβ	FaTIM1	FaACTIN	FaFHA1	FaGAPDH1	FaGAPDH2	FaCHC1	FaMT1	FaRIB413	FaUBQ1	FaEF1α	FaTUBα
	(0.84)	(0.73)	(0.72)	(0.69)	(0.64)	(0.61)	(0.59)	(0.59)	(0.53)	(0.53)	(0.46)	(0.46)	(0.40)
**FCP**	FaGAPDH1	FaFHA1	FaCHC1	FaTIM1	FaTUBβ	FaBZIP1	FaUBQ1	FaEF1α	FaACTIN	FaMT1	FaTUBα	FaRIB413	FaGAPDH2
	(0.88)	(0.73)	(0.69)	(0.66)	(0.63)	(0.63)	(0.47)	(0.47)	(0.41)	(0.38)	(0.33)	(0.30)	(0.22)
**FAP**	FaGAPDH1	FaFHA1	FaMT1	FaTIM1	FaTUBα	FaRIB413	FaUBQ1	FaEF1α	FaACTIN	FaGAPDH2	FaCHC1	FaBZIP1	FaTUBβ
	(0.65)	(0.56)	(0.50)	(0.49)	(0.46)	(0.46)	(0.43)	(0.41)	(0.34)	(0.27)	(0.27)	(0.27)	(0.24)
**HCY**	FaACTIN	FaBZIP1	FaTUBβ	FaTIM1	FaMT1	FaTUBα	FaUBQ1	FaRIB413	FaEF1α	FaFHA1	FaCHC1	FaGAPDH1	FaGAPDH2
	(0.78)	(0.73)	(0.72)	(0.60)	(0.57)	(0.56)	(0.49)	(0.48)	(0.46)	(0.44)	(0.43)	(0.40)	(0.35)
**HCC**	FaTUBβ	FaTIM1	FaACTIN	FaUBQ1	FaGAPDH1	FaMT1	FaFHA1	FaGAPDH2	FaBZIP1	FaTUBα	FaCHC1	FaEF1α	FaRIB413
	(0.83)	(0.78)	(0.75)	(0.68)	(0.67)	(0.56)	(0.56)	(0.49)	(0.44)	(0.42)	(0.38)	(0.15)	(0.15)
**All samples**	FaGAPDH1	FaBZIP1	FaTIM1	FaMT1	FaTUBα	FaCHC1	FaTUBβ	FaUBQ1	FaFHA1	FaGAPDH2	FaEF1α	FaACTIN	FaRIB413
	(1.52)	(1.36)	(1.34)	(1.26)	(1.09)	(1.06)	(0.95)	(0.89)	(0.88)	(0.85)	(0.82)	(0.76)	(0.35)

Increasing stability from left to right. STDEV and SD, represent standard deviation; Cp and Ct, represent Cq for different methods.

### Combination of All Five Methods Used for Selective Classification of Reference Genes by RankAggreg

Combined stability measurements were generated by merging all five approaches (“stability index”, geNorm^PLUS^, ΔCt method, Normfinder, and BestKeeper) to establish a consensus rank of reference genes by applying RankAggreg [40]. The input to this statistical package was a matrix of rank-ordered genes according to the different stability measurements previously computed by each of the five methods described above. RankAggreg calculated Spearman footrule distances and the software reformatted this distance matrix into an ordered list that matched each initial order as closely as possible. This consensus rank list was obtained by means of the Cross-Entropy Monte Carlo algorithm present in the software.

**Figure 4:**
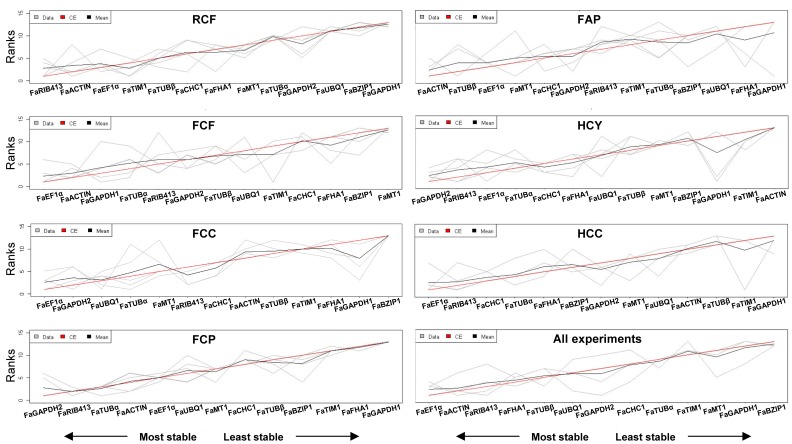
Rank aggregation of gene lists using the Monte Carlo algorithm. Visual representation of rank aggregation using Monte Carlo algorithm with the Spearman footrule distances. The solution of the rank aggregation is shown in a plot where genes are ordered based on their rank position according to their stability measurement (grey lines). Mean rank position of each gene is shown in black, as well the model computed by the Monte Carlo algorithm (red line). See Table 2 for experimental description.

As shown in Figure 4, results of the merged data revealed that the most appropriate reference genes from all the preselected candidates tested for normalization are *FaRIB413* and *FaACTIN* for analysis of strawberry fruit ripening, *FaEF1α* and *FaACTIN* for defense response studies in fruit, *FaEF1α* and *FaGAPDH2*, and *FaGAPDH2* and *FaRIB413*, for defense response studies in crown and petiole, respectively, of cultivar Camarosa, *FaACTIN* and *FaTUBβ*, for defense response studies in petiole of cultivar Andana, *FaGAPDH2* and *FaRIB413* for SA and JA treatment of in-vitro plants, and *FaEF1α* and *FaRIB413* for SA and JA treatment of cellular suspensions. Finally, *FaEF1α* and *FaACTIN* are the most stably expressed genes when all 48 experimental conditions are evaluated together.

The least stable, and therefore the least recommended reference genes are *FaGAPDH1* and *FaBZIP1* for analysis of strawberry fruit ripening, *FaMT1* and *FaBZIP1* for defense response studies in fruit, *FaBZIP1* and *FaGAPDH1*, and *FaGAPDH1* and *FaFHA1* for defense response studies in crown and in petiole, respectively of cultivar Camarosa, *FaGAPDH1* and *FaFHA1* for defense response studies in petioles of cultivar Andana, *FaACTIN* and *FaTIM1* for SA and JA treatment of in-vitro plants, and *FaGAPDH1* and *FaTIM1* for SA and JA treatment of cellular suspensions. Finally, *FaBZIP1* and *FaGAPDH1* was the least recommended when all the experiment are considered together.

### Validation of the Selected Superior Reference Genes

In order to validate the selected superior reference genes, the relative expression level of the strawberry gene encoding the transcription factor *FaWRKY1* (*AtWRKY75* ortholog, [30]) was determined in all the experimental sets of evaluated conditions. The strawberry gene *FaWRKY1* acts as positive regulator of defense response during compatible and incompatible interactions in Arabidopsis and, very likely, *Fa*WRKY1 is an important element mediating defense responses to *C. acutatum* in strawberry. We also know that the *FaWRKY1* gene is significantly upregulated in strawberry tissues under *C. acutatum* attack, and after SA and MeJA treatments ([30], Amil-Ruiz et al., unpublished data).

**Figure 5:**
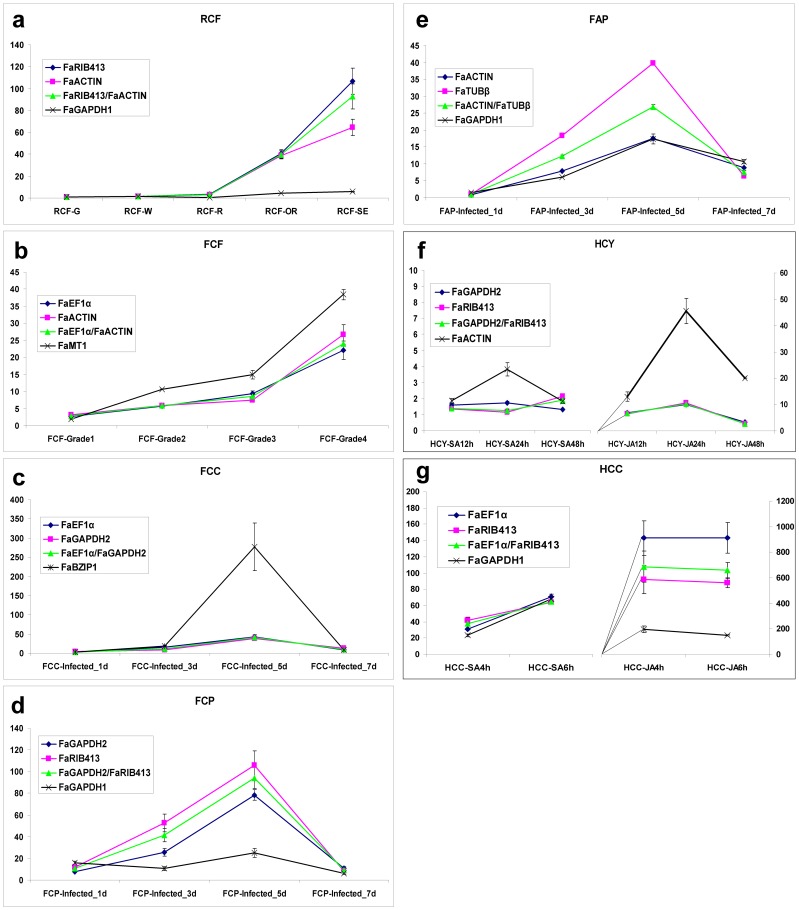
Transcript level relative quantification of the *FaWRKY1* transcription factor. *FaWRKY1* gene expression was analyzed in strawberry under the seven independent experimental conditions used in this study. Error bars show standard deviation calculated from two biological replicates. Normalization factors were calculated as the geometric mean of the expression levels of the two most stable reference genes as recommended in Figure 4 for each single experiment. Normalization to each gene individually is also shown. Additionally, the least stable reference gene was used for normalization of each experiment to demonstrate the effect of unstable reference genes in the quantification of the relative amount of target gene mRNA. Every sample was calibrated with their corresponding mock sample (see Table 2 for experimental details). Black lines linked to the X axis have been added to f and g to illustrate range of gene induction.

To analyze the bias effect on target expression analysis by selection of an inappropriate reference gene, *FaWRKY1* was normalized to either a combination of the two best candidates ranked by RankAgreg algorithm as recommended by geNorm (Figures 3 and 4), or the least recommended one. *FaWRKY1* primer sequences and other characteristics are listed in Table 1. As predicted, the reported expression profile of *FaWRKY1* is strongly affected by the choice of the reference gene. Thus, in the strawberry fruit ripening conditions (RCF) as well as for infected petioles of cultivar Camarosa (FCP) and elicited cellular suspensions (HCC), the expression level values were similar to those previously reported ([30]) when the reference genes were the two most recommended ones (*FaRIB413* and *FaACTIN*, *FaGAPDH2* and *FaRIB413*, *FaEF1α* and *FaRIB413*, respectively), either individually or combined as geometric mean (Figures 5a, 5d and 5g). To the contrary, a strong bias in the *FaWKRY1* expression pattern was obtained when the least recommended gene (*FaGAPDH1* in all three cases) was used for normalization. From these data the use of *FaGAPDH1* as reference gene somehow neutralizes the detectable induction of *FaWRKY1* during fruit ripening and senescence, in the response to infection and after elicitation with SA and MeJA compounds.

Interestingly, in other three experimental conditions (FCF, FCC and HCY) the use of the least stable reference gene (*FaMT1*, *FaBZIP1* and *FaACTIN* respectively) seemed to have opposite influence in the detection of accurate expression values of the *FaWRKY1* target gene. In this case the induction of this target gene was artificially high (Figures 5b, 5c and 5f). This is probably due to slightly but opposite variation of the corresponding reference mRNA levels during the analyzed process. These variations have significant impact in the final relative quantification of the expression of the target gene. Only ‘Andana’ petioles under fungal infection (FAP experiment) showed insignificant differences in the *FaWRKY1* expression when either the best (*FaACTIN*) or the least effective (*FaGAPDH1*) reference candidates were considered.

## Discussion

This work has mainly been focused to the evaluation of a set of potential strawberry reference genes for plant-defense response studies. Therefore, a variety of biological samples representing experimental conditions used to evaluate plant defense responses were used. The effect of natural pathogen infection as well as fruit senescence and decay are represented by experiments in this report through the analysis of fruit ripening and fruit natural infection by *C. acutatum*. Other tissues from Camarosa and Andana strawberry cultivars under fungal infection conditions were also included in this study, allowing comparisons between vegetative tissues within a cultivar, and between same tissues in different cultivars. Also, strawberry cultivars grown under contrasting contexts (in-vitro plants and cellular suspensions) were compared after treatment with either SA or JA, two phytohormones implicated in the activation of two well-known plant defense signaling pathways. A reference candidate with stability across such a range of conditions would be likely to perform well in narrower comparisons.

Several statistical procedures and software packages have been implemented to test which reference gene is best suited for transcript normalization in a given subset of biological samples. Each algorithm has its own strengths and limitations, so consensus among multiple tests provides great confidence that the results will be accurate and widely applicable.

Two methods, ΔCt and “stability index”, perform studies about the variation of ΔCt in pairwise genes or simple Ct, respectively. The comparative ΔCt method ranks the reference genes by their mean standard deviation in the pairwise comparisons, while the “stability index” approach introduces statistics and linear regression analysis to rank the candidates by the product of the coefficient of variation and slope of regression of gene means against overall means for the different samples. In the latter method (Table 3, Figure S2), although genes with the lowest “stability index” values represent the best option for normalization, many of the other strawberry candidate genes may also be considered acceptable as controls based on the SI value obtained in this study. In addition, the level of expression of the reference genes compared to that of the genes being analyzed is an important factor to be considered in certain cases [10]. Thus, the two most stably expressed strawberry genes in all seven experiments together exhibited the greatest range in steady-state transcript accumulation. *FaRIB413* was detected at relatively high levels due to its role as a structural component of the ribosome (mean Cq = 8.542), whereas *FaACTIN* was expressed at a much lower level (mean Cq = 24.011). Therefore, they may be considered as appropriate reference genes to test target genes with high or low transcript levels, respectively. Indeed, the *FaRIB413* RNA has been demonstrated to be an appropriate internal control for strawberry expression studies across several tissues and experimental conditions, using RNA-gel blots or RTqPCR analyses [41], [29], [30]. *FaRIB413* has also been recommended for studies of strawberry genes expressed at relatively low levels, but it must be diluted up to 4000 times in order to equilibrate this transcript to general expression levels an achieve comparative Cq analyses [29]. Using the “stability index” method, it appears clear that *FaACTIN* may serve as a non-diluted reference instead of *FaRIB413*.

The geNORM program (Table 4) uses pair-wise comparisons and geometric averaging across a matrix of reference genes and biological samples to determine the best reference. The program calculates the expression stability value (M_A_) and allows accurate normalization of RTqPCR data [4], [37]. However, this approach leaves the method vulnerable to errors due to co-regulation, which tends to select those genes with the highest degree of similarity in their expression profiles [9]. On the other hand, it has the advantages that it is minimally affected by expression intensity of the reference genes [42] and it can determine the optimal number of genes (V) required to accurately normalize RTqPCR data based in pairwise variation [4]. Accordingly, two common well established sets of candidates with relatively high and low stability values were detected in all experimental conditions. *FaEF1α* always appears well positioned in the experimental conditions tested, and *FaACTIN* is stably expressed in ripening and mostly all infection conditions (except in crown tissue of cultivar Camarosa).

In contrast, the *FaGAPDH1* and *FaBZIP1* transcripts mostly showed high M_A_ values (a lower stability) in all conditions. *FaFHA1* is stably expressed in all conditions except in all infected tissues from cultivar Camarosa, and *FaRIB413* is also stable but only in infected crown and petiole tissues from the same cultivar. On the other hand, the *FaTIM1* transcript presented high M_A_ values in all conditions except the two fruit experiments, where its accumulation was stable. The *FaMT1* transcript presented low stability in all ‘Camarosa’ experimental conditions, but low M_A_ values when cultivar Andana and Chandler are considered.

Unlike geNORM, NormFinder is not affected by correlated expression of the candidate genes (Table 5). However, the latter gains in robustness as the sample number is increased, while geNorm doesn’t need large sample size since it uses pair-wise comparisons. The Bestkeeper algorithm also performs pairwise comparisons using the geometric mean of the Cp (Cq) values, but different expression levels can generate heterogeneous variance between groups, and this can invalidate the use of Pearson correlation coefficient [43], [5]. The results from these two methodologies coincide with that of ΔCt method, and taken together these results indicate that gene *FaEF1α* seemed to be the most stably expressed reference gene meanwhile genes *FaGAPDH1* and *FaBZIP1* were the least stable ones.

### Recommended Reference Genes in a Strawberry-defense Response Context

We have applied RankAggreg [40] to establish a consensus rank of reference genes by combination of all five above methods. This approach strengthens the value of the recommended candidates to normalize target gene expression in any of the conditions here described. Thus, results in Figure 4 show genes recommended in each particular experiment, suggesting they can be used as superior reference genes for transcript quantitation. Taken together, we propose genes *FaRIB413*, *FaACTIN*, *FaEF1α* and *FaGAPDH2* as superior reference genes for accurate transcript normalization in strawberry (*Fragaria × ananassa*) under the described experimental conditions.

The genes proposed here have been reported in previous strawberry studies (see Table 1), although no experimental work was performed to validate their usefulness as RTqPCR reference genes in a variety of tissues, treatments or conditions. As previously stated, the *FaRIB413* gene has been extensively used for northern and RTqPCR normalization in strawberry [41], [29], [44], [30], [45], [46]. However, *FaRIB413* encodes a highly abundant ribosomal RNA (Cq around 8 in our study, Table 3), which does not contain a poly(A) tail, making it unsuitable for RTqPCR analysis aimed at differentiating the expression levels of rare genes, and also for the synthesis with cDNA using oligo(dT) primers. Although *FaRIB413* presents good values of expression stability in almost all of the experiments analyzed by RankAggreg (Figure 4), it is strongly recommended that an alternative strawberry reference with Cq values as close as possible to the Cq values showed by the target gene be used.

An actin transcript was used by Lin-Wang et al. (2010) [28], for normalization of RTqPCR studies in different strawberry plant tissues. The authors selected this gene as a reference gene “*because of its consistent transcript level throughout fruits and leaves*”. From our results, *FaACTIN* presents high stability in all fruit experimental conditions, such as ripening and infection, in ‘Andana’ petiole tissues, and also considering all the experiments together. These data match well with the analysis reported by Lin-Wang et al. (2010) [28]. However, this *FaACTIN* gene was not appropriate when vegetative tissues of cultivar Camarosa (crown and petioles) were exposed to fungal infection, or by phytohormone elicitation either of strawberry plants or cellular suspensions.

Also, a strawberry elongation factor 1α gene (*EF1a*) was used by Guidarelli et al. (2011), to normalize raw expression data in an RTqPCR experiment with fruits of the very susceptible strawberry cultivar Alba inoculated with *C. acutatum*. [27]. Although authors did not assess the stability of expression of this gene by none of the available methods, they detected that this gene had “*the most constant expression levels (absolute ΔCt <1 among treatments)*”, and assumed this candidate gene for data normalization. From our results, *FaEF1α* is indeed recommended as the best candidate for normalization of experiments based on strawberry fruits under biotic interaction. Therefore, our analysis agrees with the controls used by Guidarelli et al. (2011).

In addition, *FaGAPDH1* and *FaGAPDH2* genes have been previously used as reference genes in plant-pathogen interaction studies [26], [47], [48], [49]. In the case of *FaGAPDH2* gene reported by Khan et al. (2004), our results support the use of this gene as control in the experimental conditions reported by these authors, (i.e. strawberry vegetative tissues inoculated with *Colletotrichum*) (see Figure 4). The *FaGAPDH1* reference gene has been reported for use in strawberry experimental treatments with phytohormones or after fungal inoculation, as reported Grellet-Bournonville et al. (2012), Mamaní et al. (2012) and Zamora et al. (2012). The data in the current report indicate that this reference may not have been the best choice as this transcript has shown the lowest values of stability in almost all the experimental conditions.

The comparative analysis between using the most and the least appropriate reference gene in a given experiment (Figure 5) illustrates the magnitude of the bias produced by normalization with an unstable gene, and also highlights how the incorrect use of reference genes without any previous validation can lead to misinterpretation of data. For this reason we strongly recommend to perform a validation of the putative reference genes prior any quantitative expression studies, as recommended elsewhere [50], [23], [51], [52], [19].

It is important to note that in certain species even the best reference candidates show some variation across the different tissues, developmental stages and environmental conditions [10]. Differences in the defense gene expression patterns have been reported across different strawberry tissues and cultivars challenged with *C. acutatum* [29]. These observations indicate that the first step in any gene expression experiment should be to test reference candidates in the specific genetic background and in the same experimental conditions. This validation is especially important when testing effects of strong biotic or abiotic stresses, such as pathogen challenge.

In conclusion, stably expressed genes were selected from two independent strawberry biological replicates of a total of forty eight samples, representing seven different experimental conditions. Our results represent a relevant contribution to the scientific plant community as the best candidates for reference genes in strawberry. The candidates have been ranked accordingly to their respective expression stability in a variety of samples representing major conditions typically used in a plant-defense context. The identification of other stable reference pools under different experimental conditions would build a useful community resource for gene expression analysis in this crop.

## Materials and Methods

### Plant Materials and Growth Conditions

Plant material, *Fragaria × ananassa* cultivars Chandler, Camarosa and Andana were used. *Colletotrichum acutatum*, a major strawberry pathogen was used for natural infection and controlled inocculation. All the plant culture and growth conditions, *C. acutatum* experimental conditions, and treatments with chemicals have been previously described [29], [30], and are summarized in Table 2. Briefly, strawberry cellular suspensions (cv. Chandler) were prepared from *in vitro* growing calli. Five days old cell suspensions were treated with MeJa (0.1 mM), SA (0.75 mM) or water (as control). Alicuots were taken at 2 hour intervals and cells were frozen in liquid nitrogen. Samples at 4 and 6 hours were used in this work because they match with a strong relative expression of the *FaWRKY1* target gene, and many other strawberry genes currently under study in our lab. Axenic *in-vitro* plants from cv. Camarosa were aseptically sprayed with water, MeJa (2 mM) and SA (5 mM) solutions and collected at 12, 24 and 48 hours post-treatment. Strawberry fruits were collected from a growing field in several ripening stages and pooled by stage. Red stage strawberry fruits naturally-infected by *Colletotrichum acutatum* and exhibiting different increasing degrees of fungal necrotic lesions were collected and fruits having similar symptoms were pooled. No specific permissions were required for these activities. None human manipulation was applied to strawberry field prior to sample collection. Field studies did not involve endangered or protected species. Eight-week-old strawberry plantlets were placed in 20 cm diameter plastic pots containing sterilized peat and grown for a minimum of six additional weeks prior to mock or pathogen inoculation by spraying a spore suspension of 10^6^ CFU ml^−1^. Crowns and petioles were collected 1, 3, 5 and 7 days after treatment. All samples were flash frozen in liquid nitrogen and stored at −80°C until needed.

### RNA Preparation for RTqPCR

Total RNA from strawberry fruits and vegetative tissues, as well as cell suspension cultures, was isolated according to Manning [53], treated with DnaseI (Invitrogen) to remove the residual contaminating DNA, and further purified with the RNeasy MinElute Cleanup Kit (QIAGEN). Extracted RNA samples showed high degree of purity, without residual contamination by organic compounds, accordingly to Accerbi et al. (2010) [54]. RNA samples were tested to be free of genomic DNA contamination after DNase I treatment by performing a qPCR analysis using the primer pairs corresponding to the *FaGAPDH2* and *FaRIB413* genes. Amplicons corresponding to these two genes were undetectable in the RNA samples after 40 cycles as confirmed by qPCR or by agarose gel electrophoresis (data not shown). These results indicated that amplicons generated by RTqPCR analysis were produced only from synthesized cDNA. Purified RNA was quantified by the NanoDrop 1000 Spectrophotometer (Thermo scientific) and the integrity checked by agarose gel electrophoresis and the Agilent 2100 Bioanalyzer (Agilent Technologies, Deutschland). All the samples showed RIN values over 8 (data not shown) and therefore were deemed suitable for RTqPCR analysis.

To ensure equal concentrations of RNA in all samples prior to the RT reactions, samples were diluted to 200 ng/ul and reassessed three times in a serial dilution of 1∶0, 1∶5 and 1∶25, to ensure fidelity of the measure. First-strand cDNA synthesis was carried out by the iScript cDNA Synthesis kit (Bio-Rad) using as template 1 µg of purified total RNA per 20 µL of reaction volume. RT reactions were diluted 5-fold with nuclease-free water prior to be used in the qPCR.

### Real-time qPCR

Specific primer pairs set for the genes tested were designed using Oligo Primer Analysis software version 6.65, tested by dissociation curve analysis, and verified for the absence of non-specific amplification. More details are provided in results. RTqPCR runs were performed in MyIQ and iCycler real-time PCR systems (Bio-Rad) using 96-well plates and 20 µL final reaction volume per well. Two µL template cDNA was added to the PCR reaction mixture containing 0.4 µM of each primer and 10 µL of 2× SsoAdvanced™ SYBR® Green supermix (Bio-Rad). The protocol was: an inicial step of enzyme activation/DNA denaturation of 95°C for 1 min, followed by 40 cycles of 95°C for 15 sec, 65°C for 15 sec and 72°C for 15 sec, and a final standard dissociation protocol to obtain the melting profiles. Data were adquired by means of the MyIQ v1.004 and iCycler v3.1 softwares (Bio-Rad).

### Computational Data Analysis

Data analysis strategy is described in detail in the results section. Reaction efficiency calculus was done using LinRegPCR version 2012.3 [36], [55]. Resulting mean PCR efficiencies per amplicon were taken. Reference genes validation was performed using previously described software applications, included the MS Excel VBA applets NormFinder v0.953 [9] and BestKeeper v1 [38], and the geNorm [4] algorithm pr**ov**ided in qBasePlus v2.4 package [37]. Other statistical procedures were performed with the free software R v2.15.2 (http://www.R-project.org), with the packages RankAggreg 0.4–3, clValid 0.6–4 and gtools 2.7.0; and SPSS software ver 15.0 for Windows.

## Supporting Information

Figure S1 **Dissociation curves and agarose gel analysis of the amplicons tested in this study.** (a) Melting curve analysis of 13 potential reference genes along with control gene for validation (*FaWRKY1*) was carried out to confirm the absence of multiple amplicon species after RTqPCR. Each line represents a melting curve of amplicons from two technical replicates of two biological replicates in the given experiments. (b) Agarose gel electrophoresis of RTqPCR products after 40 cycles of PCR.
(TIF)

Figure S2 **Regression analysis for several genes showing predicted regression lines and actual means over all experiments.** The most stable and consistent control genes would have the lowest slope and closest fit to the regression line. (a) *FaACTIN* (first in top) had the highest stability and *FaRIB413*, as well as *FaEF1α* and *FaTUBβ*, have also very good values of stability (from first in bottom to second in top). (b) Genes *FaBZIP1* and *FaTIM1* had the lowest stability index. See Table 2 for descriptions of tissue samples, represented here by abbreviations.
(TIF)
