# Supplementary figures and images for: Identification and Validation of Reference Genes for Transcript Normalization in Strawberry (Fragaria × ananassa) Defense Responses
Source: PLoS One. 2013 Aug 5;8(8):e70603. doi: 10.1371/journal.pone.0070603 (PMC3734262; doi:10.1371/journal.pone.0070603)

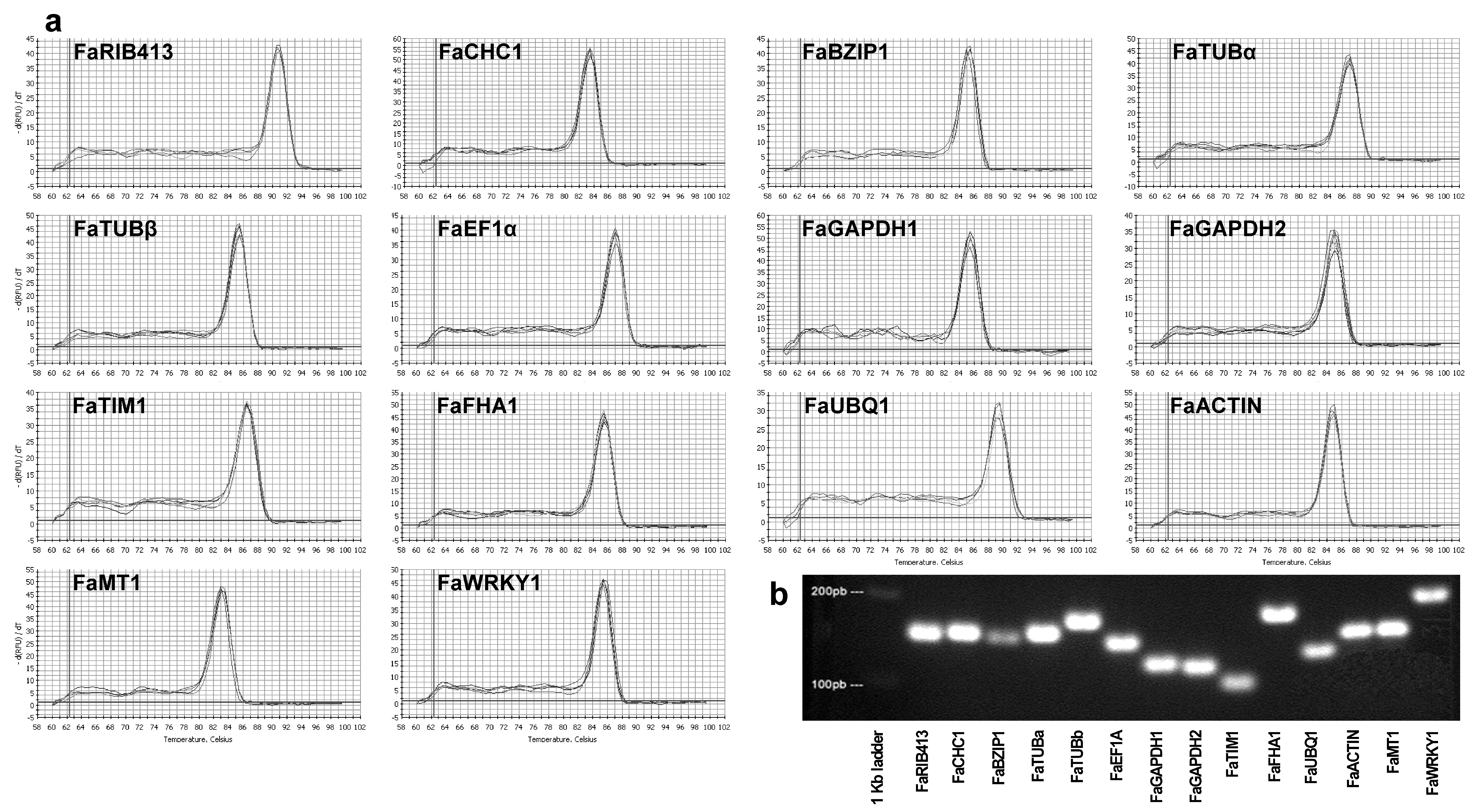

Supplement: Figure S1 — Dissociation curves and agarose gel analysis of the amplicons tested in this study. (a) Melting curve analysis of 13 potential reference genes along with control gene for validation (FaWRKY1) was carried out to confirm the absence of multiple amplicon species after RTqPCR. Each line represents a melting curve of amplicons from two technical replicates of two biological replicates in the given experiments. (b) Agarose gel electrophoresis of RTqPCR products after 40 cycles of PCR. (TIF) [file pone.0070603.s001.tif]

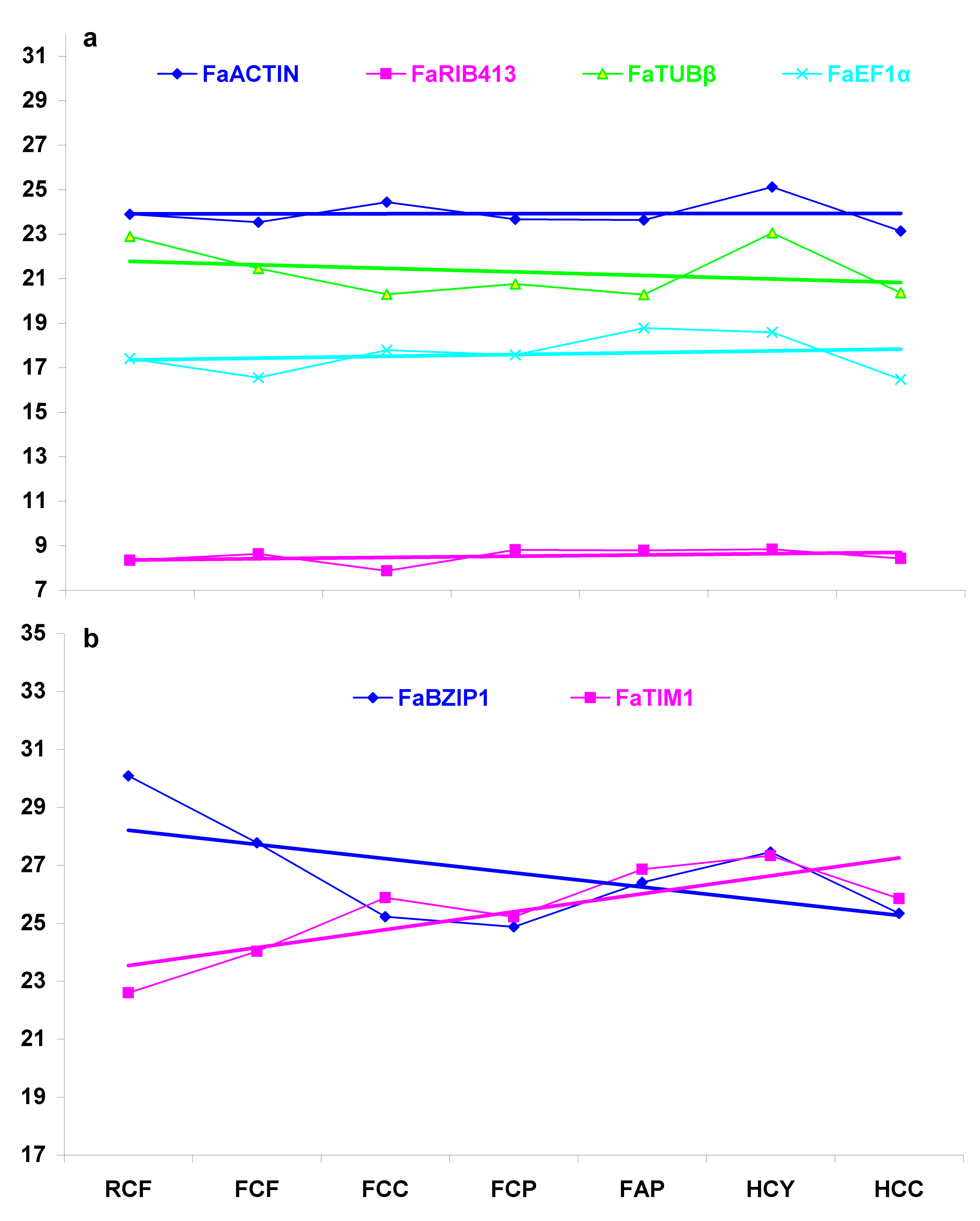

Supplement: Figure S2 — Regression analysis for several genes showing predicted regression lines and actual means over all experiments. The most stable and consistent control genes would have the lowest slope and closest fit to the regression line. (a) FaACTIN (first in top) had the highest stability and FaRIB413, as well as FaEF1α and FaTUBβ, have also very good values of stability (from first in bottom to second in top). (b) Genes FaBZIP1 and FaTIM1 had the lowest stability index. See Table 2 for descriptions of tissue samples, represented here by abbreviations. (TIF) [file pone.0070603.s002.tif]
